# Guinea worm in domestic dogs in Chad: A description and analysis of surveillance data

**DOI:** 10.1371/journal.pntd.0008207

**Published:** 2020-05-28

**Authors:** Sarah Anne J. Guagliardo, Sharon L. Roy, Ernesto Ruiz-Tiben, Hubert Zirimwabagabo, Mario Romero, Elisabeth Chop, Philippe Tchindebet Ouakou, Donald R. Hopkins, Adam J. Weiss

**Affiliations:** 1 Parasitic Diseases Branch, Division of Parasitic Diseases and Malaria, Centers for Disease Control and Prevention, Atlanta, Georgia, United States of America; 2 Guinea Worm Eradication Program, The Carter Center, Atlanta, Georgia, United States of America; 3 Guinea Worm Eradication Program, Ministry of Public Health, N’Djamena, Chad; Makerere University, UGANDA

## Abstract

After a ten-year absence of reported Guinea worm disease in Chad, human cases were rediscovered in 2010, and canine cases were first recorded in 2012. In response, active surveillance for Guinea worm in both humans and animals was re-initiated in 2012. As of 2018, the Chad Guinea Worm Eradication Program (CGWEP) maintains an extensive surveillance system that operates in 1,895 villages, and collects information about worms, hosts (animals and humans), and animal owners. This report describes in detail the CGWEP surveillance system and explores epidemiological trends in canine Guinea worm cases during 2015–2018. Our results showed an increased in the number of canine cases detected by the system during the period of interest. The proportion of worms that were contained (i.e., water contamination was prevented) improved significantly over time, from 72.8% in 2015 to 85.7% in 2018 (Mantel-Haenszel chi-square = 253.3, P < 0.0001). Additionally, approximately 5% of owners of infected dogs reported that the dog had a Guinea worm-like infection earlier that year; 12.6% had a similar worm in a previous year. The proportion of dogs with a history of infection in a previous year increased over time (Mantel-Haenszel chi-square = 18.8, P < 0.0001). Canine cases were clustered in space and time: most infected dogs (80%) were from the Chari Baguirmi (38.1%) and Moyen Chari Regions (41.9%), and for each year the peak month of identified canine cases was June, with 78.5% occurring during March through August. Findings from this report evoke additional questions about why some dogs are repeatedly infected. Our results may help to target interventions and surveillance efforts in terms of space, time, and dogs susceptible to recurrent infection, with the ultimate goal of Guinea worm eradication.

## Author summary

Guinea worm is a parasitic infection caused by the roundworm *Dracunculus medinensis*. The potential route(s) and risk factors for transmission in dogs are under research. Since 2012, the Chad Guinea Worm Eradication Program (CGWEP) has conducted intensive surveillance to detect infections in dogs in addition to ongoing surveillance to detect cases in humans. In this report, we describe the methods employed by CGWEP to identify canine cases and explore the characteristics of infected dogs. During the period of interest, 2015–2018, there were an increased number of canine cases detected by the system, with evidence of better canine case containment over time (i.e., contamination of water was prevented). About 13% of dogs had experienced a previous Guinea worm infection in a different year, suggesting that infection risk is not equally distributed among all dogs over time. Two regions within Chad, Chari Baguirmi and Moyen Chari Regions, reported approximately 80% of all canine cases detected, and a strong seasonal pattern was observed.

## Introduction

Since the inception of the global Guinea Worm Eradication Program in 1980, the number of human cases of Guinea worm disease declined from an estimated 3.5 million cases in 1986 in 21 countries to fewer than 30 cases in only five countries in 2018 [1–3]. (South Sudan seceded from Sudan in 2011; in 1986 there were 20 countries reporting Guinea worm cases).

*Dracunculus medinensis* is the causative agent of Guinea worm, and human infection occurs upon consumption of water containing copepods (miniscule freshwater crustaceans) that are infected with *D*. *medinensis* larvae. During the course of approximately 10 to 14 months after infection, the male and female worms mate inside the host, and the pregnant adult female worm migrates through the subcutaneous tissues and toward the skin surface [4]. As the female worm begins to emerge, a painful burning blister or lesion forms on the skin, usually on a distal lower extremity. When the emergent worm comes into contact with water, larvae are released, which are then ingested by a copepod, beginning the cycle again [4]. It is thought that development of the parasite in dogs occurs over a similar timeframe as observed in humans. Several historical experimental studies on dogs and rhesus monkeys [5, 6] note that time from infection to worm maturity ranges from 10–14 months [7].

The Chad Guinea Worm Eradication Program (CGWEP) in the Republic of Chad currently reports the majority of *D*. *medinensis* infections in humans, dogs, and cats worldwide [1, 2]. After an apparent ten-year absence of Guinea worm in Chad, human cases were rediscovered in 2010; canine cases were first recorded in 2012 [8]. Since 2012, the majority of *D*. *medinensis* infections in Chad occur in canine hosts [8], representing the first observation of sustained transmission of the parasite in dogs in Africa. Historical accounts have documented canine cases in other regions of the world, including southern India, Uzbekistan, Azerbaijan, Kazakhstan, and possibly Turkmenistan (where Guinea worm was not thought to be endemic) [9–15]. In areas where infections in both humans and dogs occurred, dog infections historically decreased simultaneously with decreasing human cases as a result of introduced control measures [7, 16]. Yet in some regions (i.e., Bukhara, Uzbekistan), canine cases curiously persisted even after human cases declined, albeit for a short period of time [15]. The eco-epidemiological patterns of transmission in Chad are similarly puzzling. Eberhard et al noted that, in comparison with past epidemiological trends, cases in humans appear to be sporadic, with no clustering by village, and no apparent association with common water sources [8]. This unusual transmission pattern, in conjunction with the persistence of infections in dogs, raises the possibility of a deviation from the typical *D*. *medinensis* life cycle. It is hypothesized, for instance, that a paratenic or transport host is involved, such as fish or frogs [17]. Comprehensive information about the descriptive epidemiology in dogs could help to both direct the future Guinea worm research agenda and to provide insights as to how the program might more efficiently target education efforts. If, for example, there was evidence of recurrent infection in some dogs, it might prompt the program to conduct more rigorous tracking of these animals over time.

In response to the rediscovery of human cases in 2010, the Chadian Ministry of Public Health (MOPH) requested support from The Carter Center to revitalize its national Guinea Worm Eradication Program. In 2012, the CGWEP was re-established, beginning with active surveillance in 632 villages located within the catchment areas of health centers that reported human cases in 2010–2011 [18]. Upon the discovery of more human cases and canine cases, the program expanded coverage to include more villages each year—at the end of 2018, 1,895 villages were included in the active village-based surveillance system [19]. Both passive and active surveillance approaches operate in Chad, with active surveillance areas operating in those areas thought to be at greatest risk for transmission. But there are questions about the relationship between surveillance system intensity and canine case finding. For example, does the surveillance system adequately contain cases that are detected at all levels of surveillance? (In other words, is water contamination prevented?).

The purpose of this report is to describe the active surveillance system used to identify canine cases, evaluate surveillance outcomes (e.g., case containment) by surveillance level to assess program trends, and identify common case characteristics and potential risk factors for disease to generate hypotheses for future research.

## Methods

### Ethics statement

Data collected for this analysis is part of routine public health surveillance conducted by the Chadian Ministry of Public Health. Analysis of Guinea worm surveillance data was given a non-research determination by the delegated authority at the U.S. Centers for Disease Control and Prevention Center for Global Health (project ID: 0900f3eb819c65ad).

### Surveillance system

**Fig 1:**
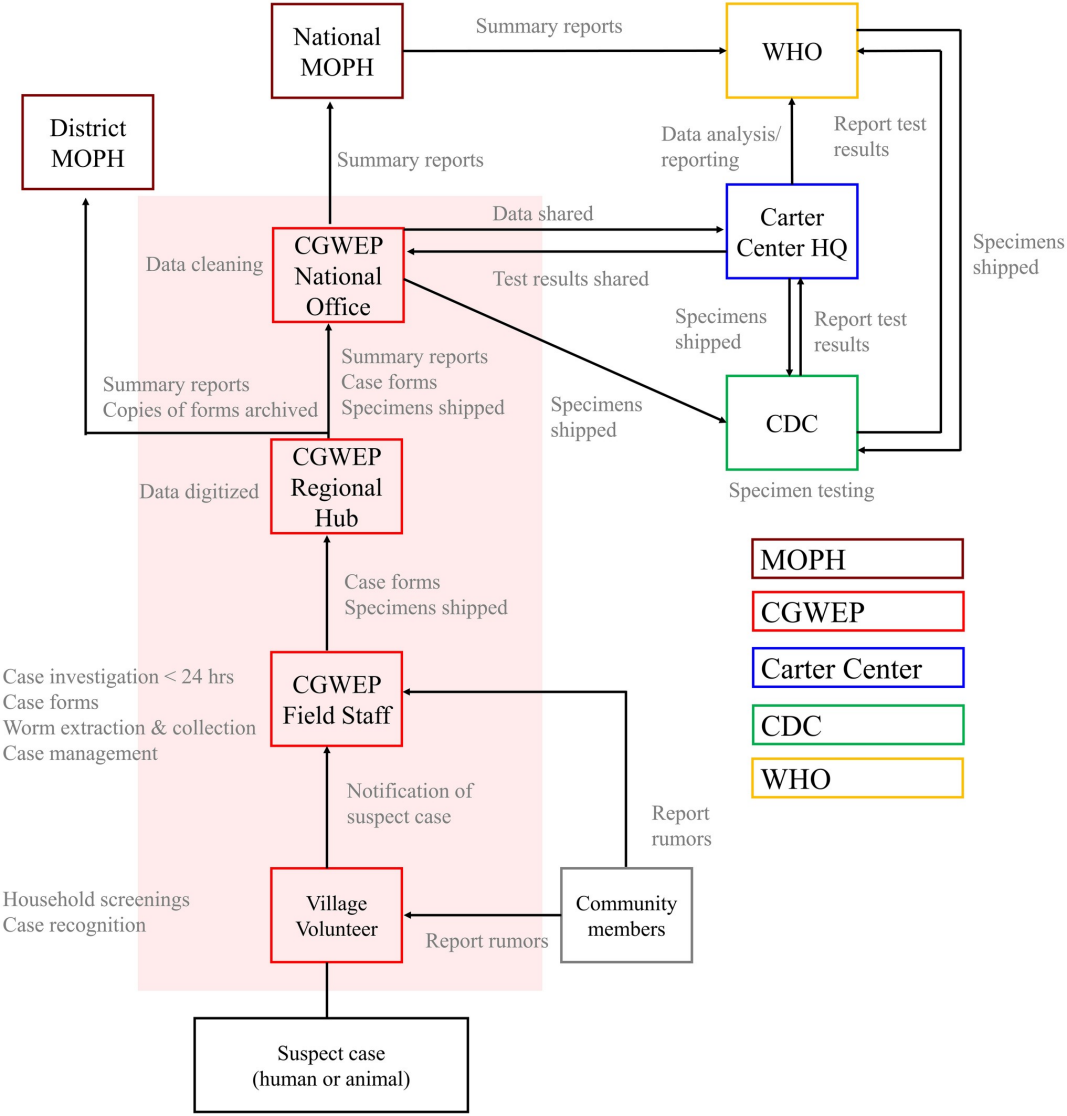
Chad Guinea Worm Eradication Program surveillance system (CGWEP). The active surveillance system (Levels 1 and 2) is summarized by the figure above. The CGWEP (red boxes and red shaded area) is housed within the Chad Ministry of Public Health (MOPH) (maroon boxes).

The CGWEP operates at three different levels of tiered intensity that correspond to risk of transmission (i.e., the number of cases observed in the past), including two levels of active surveillance and one level of passive surveillance (Fig 1, Table 1). All levels of the surveillance system rely on a cash reward program to provide financial incentives for community members to report rumors of suspect cases in humans and animals [8]. In 2015–2018, a dog owner received 10,000 Central African Francs (about $17 USD) and three bars of soap for a reporting a Guinea worm-infected dog prior to worm emergence (see S1 Table for more detail). CGWEP field staff periodically evaluate the degree of awareness of the cash reward system and level of knowledge about Guinea worm; a hotline also exists to facilitate the reporting of rumors. The cash reward amounts are consistent for all areas of Chad.

**Table 1:** Chad Guinea Worm Eradication Program surveillance levels.

	Surveillance Level		
	Level 1	Level 2	Level 3
	Health zones reporting cases (humans/animals)	Zones that have reported only few cases,* and that are in geographic proximity to Level 1 zones	Zones without cases, and that are in geographic proximity to Level 2 zones
Type of surveillance	Active	Active	Passive
Additional supervision unit^†^	Yes	—	—
Household screenings	Yes	Yes	—
Training/evaluation of CGWEP staff	Yes	Yes	—
Rumors investigated within 24 hours of notification	Yes	Yes	Yes
Worm extraction	Yes	Yes	Yes
Selective specimen testing at CDC	Yes	Yes	Yes
Cash reward system	Yes	Yes	Yes
Information campaign^‡^	Yes	Yes	Yes
Assessment of cash reward awareness	Yes	Yes	Yes
Evaluation of Guinea worm knowledge	Yes	Yes	Yes

*Approximately < 10 cases per zone.

^†^The additional supervision unit consists of village-level supervisors, who receive technical training and are supported by the CGWEP.

^‡^Health education efforts are conducted during household visits (Levels 1 and 2); town criers deliver Guinea worm-related information at weekly, centralized markets (primarily Levels 1 and 2, but also in Level 3); mass communications campaigns (all levels of surveillance in southern provinces) consist of posters placed in public locations, radio announcements, television commercials, and theatre groups that present sketches of Guinea worm and what to do if Guinea worm is encountered. Community based education efforts are also carried out at public gatherings (i.e., schools, religious centers, soccer matches, etc).

**Fig 2:**
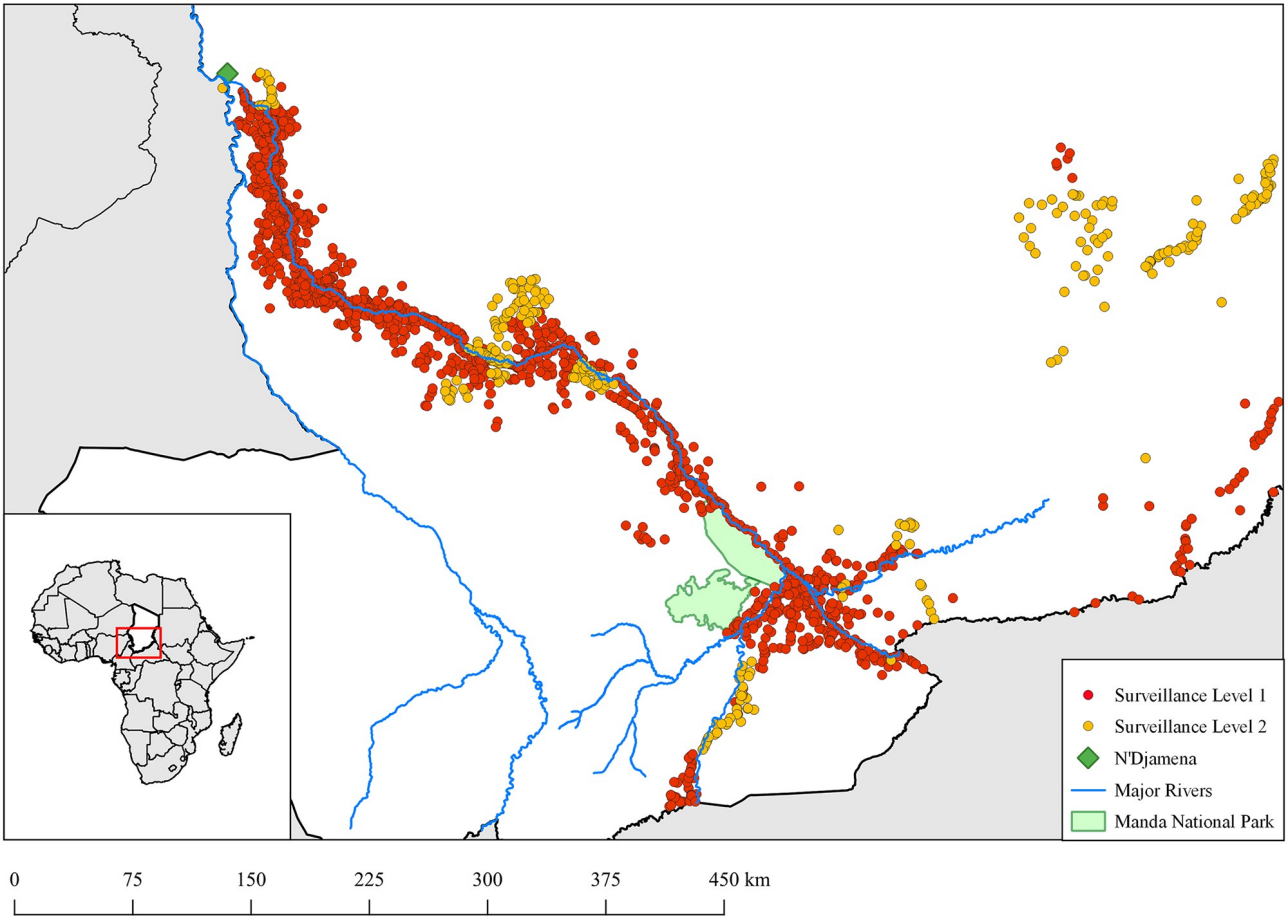
Villages under active surveillance in 2018. In 2018 there were approximately 1,900 villages under active surveillance (including Levels 1 and 2). Most of these villages are distributed along the Chari River, where most human and canine cases are thought to occur.

Level 1 includes zones where transmission has occurred consistently since 2010—essentially those areas located near the Chari River and its tributaries (Fig 2). At this level, village volunteers (usually two per village) conduct household searches (each house is visited 3–4 times per week) for people or animals with signs or symptoms consistent with Guinea worm. Volunteers are incentivized by participation in trainings, and through the receipt of t-shirts, backpacks, and health education materials. CGWEP supervisors are notified and rumors are investigated as quickly as possible, ideally within 24 hours of notification (see ‘Containment Criteria’ below). Supervisors routinely oversee field staff to ensure the quality and timeliness of surveillance activities, and to educate staff to improve knowledge of Guinea worm, surveillance, and the reward system. In recent years, some highly endemic villages have implemented an additional volunteer program, the ‘dog police’, engaging 8–15 year old children in searching for and reporting dogs with signs of Guinea worm. (See S1 Appendix for further detail about community involvement and incentives for volunteers). Communities under Level 2 surveillance are non-endemic areas in which very few cases have been reported (≤ 10 per zone), likely as a result of importation from Level 1 areas. Surveillance Level 2 includes zones adjacent to Level 1 zones; residents in Level 2 areas may share with Level 1 areas common roads/pathways and water sources. Level 2 residents may also work in Level 1 zones. Level 2 surveillance is similar to Level 1, except that there is less supervision (Table 1). Lastly, Level 3 is the least robust of the surveillance tiers and relies on passive reporting through MOPH infrastructure. Level 3 areas are those that have not reported any cases but remain at risk of importation of human or animal cases from other areas. Rumors are reported to MOPH health center staff, who in turn report rumors up the chain to the zone (catchment area for health centers), district (consisting of health zones), province (consisting of districts), and national levels [20].

Reports of investigated human and animal cases are shared with the MOPH at the district, provincial, and national levels (Fig 1). Paper case investigation forms are transported to regional CGWEP hubs, where the data are entered into Microsoft Excel spreadsheets. Quality control checks are conducted on the aggregated data; the data are shared monthly with The Carter Center headquarters in Atlanta for further cleaning and analysis. Reports and data summaries are also shared with the World Health Organization (WHO), the Centers for Disease Control and Prevention (CDC), and other stakeholders.

### Confirmatory testing

**Fig 3:**
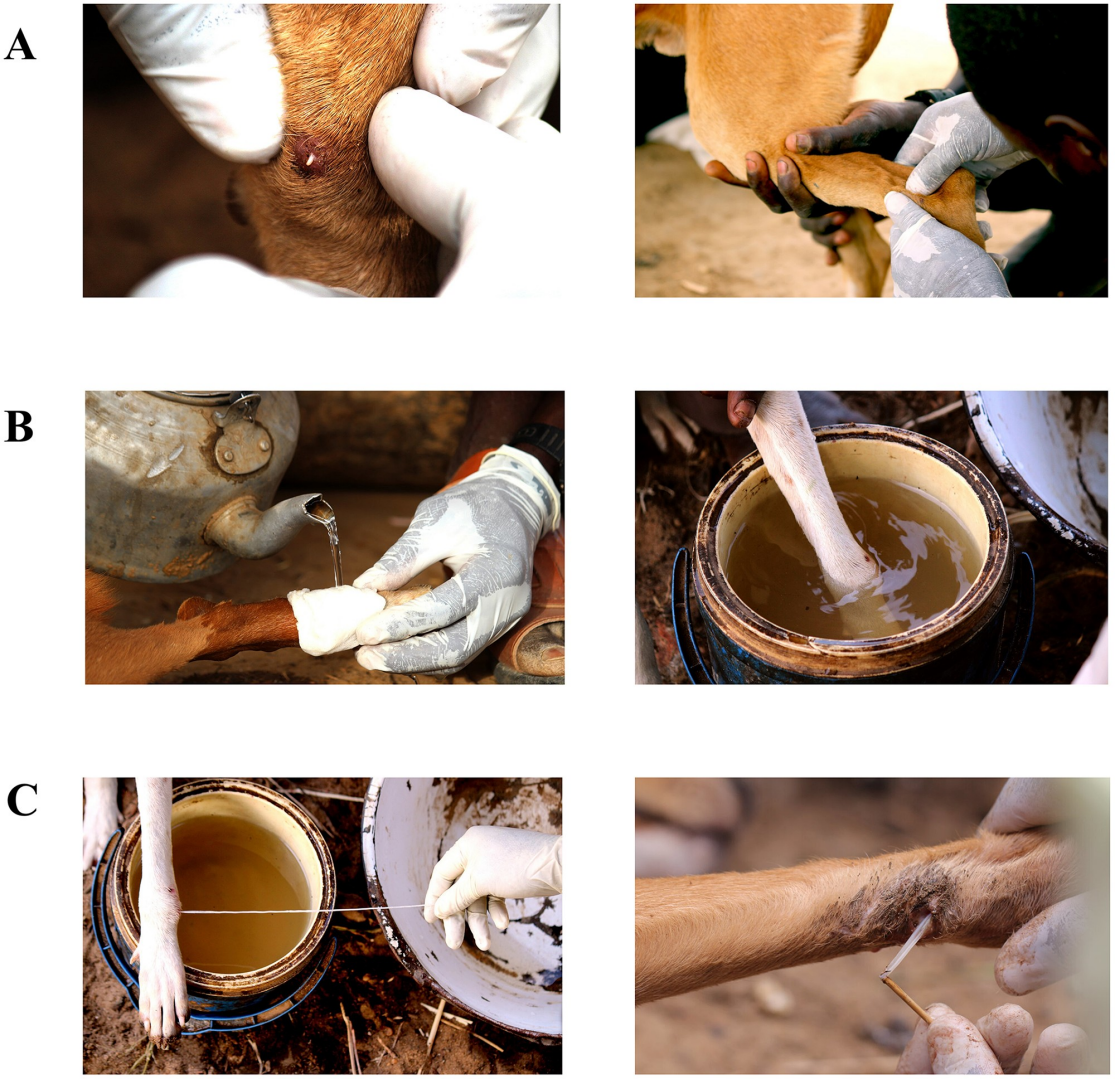
Worm extraction in dogs. The identification of pre-emergent worms (A) is challenging because blisters and worms are often obfuscated by dog fur. The “controlled immersion” technique (B) is performed by pouring water over the lesion (left) or submerging the lesion in a bucket of water (right) to allow female adult worms to release larvae within a contained environment. This water is then safely disposed of (e.g., poured on the ground away from any water sources). The worm is finally extracted (C) by gently pulling on the worm (left) or by wrapping it around a wetted gauze. A small stick is also sometimes used to initiate the process (right). Worm extraction in people can range from one day to 2–3 weeks [7, 22], but CGWEP field staff have reported that worm extraction in dogs is typically quicker than in humans. (Photo credits: Robert Hartwig, The Carter Center.)

At all surveillance levels, CGWEP and MOPH workers manually extract worms from both human and animal hosts for laboratory confirmation (see Fig 3 and S1 Appendix). Collected worm specimens are visually inspected by CGWEP field staff and are preserved in ethanol for testing at the CDC Parasitic Diseases laboratory in Atlanta, Georgia. All worms extracted from humans are sent for confirmatory testing, but worms extracted from animals are tested selectively (e.g., occurrence of dog infections from an area previously thought to be Guinea worm-free). (Although early in the program’s history worms extracted from any host were sent for confirmatory testing, it was decided in 2014 to only test select worms emerging from dogs. This was due to the large volume of worms extracted from dogs, and because of the observation that the majority of worms tested from dogs were *D*. *medinensis*.) At CDC, worm specimens initially undergo morphological examination, and when microscopy results are ambiguous, polymerase chain reaction (PCR) tests are conducted for speciation [21]. Test results are reported back to The Carter Center headquarters, WHO headquarter and regional offices, and the in-country point-of-contact to disseminate to the field level. If a specimen is thought to be *D*. *medinensis* by the CGWEP field staff, interventions are implemented per current program policy immediately, without waiting for laboratory confirmation. CGWEP defines a canine case as a dog with a worm that has been verified as Guinea worm by a supervisor (but that has not necessarily been laboratory-confirmed).

### Data collected

The CGWEP collects information about infected dogs through a standard survey with the self-identified dog owner, most commonly the head of household. Canine demographic variables collected include the dog’s estimated age (months, as reported by the dog owner), sex, location (village, and if applicable, neighborhood), and the primary use(s) of the dog (i.e., whether it is used for hunting or guarding). Owners are interviewed about places the dog frequents to inform decision-making about temephos applications (an insecticide) to specific bodies of water. CGWEP also collects information from dog owners on the number of dogs residing in the household, whether the owner has had other infected dogs, and owner ethnicity and occupation. Further information is collected about the worm that emerged, the lesion location on the dog’s body, the date the worm was reported and visually inspected, and whether all case containment criteria were satisfied. These worm-level variables are captured each time a worm emerges, whereas dog-level information is collected only once. Village-level information is collected through observation by asking village leadership about whether the village is primarily a fishing village (close proximity to water, in which the majority of families fish), and whether the village has access to at least one potable water source (e.g., at least one borehole well with a functional pump). A copy of the 2018 surveillance form used to collect this information is included in S2 Appendix.

### Canine case containment criteria

An adult Guinea worm extracted from a dog is considered to be “contained” when all of the following conditions are met:

1. The rumor of the dog infection is investigated within 24 hours; and
2. The dog has not entered any water source since the worm emerged; and
3. The dog was tethered until all signs and symptoms of infection have fully healed (to prevent contamination of water sources); and
4. The containment process, including (visual) verification the case is Guinea worm disease, is validated by a supervisor within 7 days of the emergence of the worm.

An infected dog is considered to be contained if all of the emerging Guinea worms associated with that dog were contained. Since 2012, the dog case-containment criteria have changed from year to year, and have evolved to become more specific over time. The criteria above are generalized, allowing for comparisons to be made on a yearly basis. For example, in years past the criterion for tethering the dog did not specify *how* the dog was tethered, and in 2018, the criterion was slightly modified to state that dogs must be tethered with a lock and chain. CGWEP paid field staff and volunteers work to educate and encourage dog owners to provide both food and water to dogs for the duration of tethering.

### Data management and analysis

Data management and analyses were conducted in SAS 9.3 (Cary, North Carolina) and graphs were developed in the R base package [23]. Maps were developed in Quantum GIS [24], and shapefiles of key landscape features (Chari River, Manda National Park) were extracted courtesy of Landsat imagery available from the U.S. Geological Survey [25].

We analyzed data in terms of (i) descriptive characteristics of worms and infected dogs (ii) descriptive characteristics of owners of infected dogs, (iii) spatial and temporal distribution of infected dogs. The proportions of worms and dogs contained for each year were calculated by surveillance level as a possible way to evaluate surveillance system response strength at these different levels (Mantel-Haenszel chi-square, P < 0.05). The age of infected dogs by year and region were compared (Kruskal-Wallis, P < 0.05). We also calculated the annual proportion of dogs with a history of Guinea worm (reported by the owner) within the same year of detection or in a past calendar year (Mantel-Haenszel chi-square, P < 0.05). The proportion of infected dogs that derived from different regions was calculated and mapped, and the raw number and cumulative canine cases by month and year were graphed. To display seasonal trends over space, graphs of canine case counts by region and month were also developed.

## Results

### Characteristics of worms and infected dogs

In total, 6,348 Guinea worms were reported from infected 3,371 dogs from 2015 through 2018. Of these, 13 were laboratory confirmed as *D*. *medinensis* (10 confirmed by morphology, and 3 confirmed by PCR) (S2 Table, also partially reported in [1, 2, 26]). The proportion of worms that were contained was significantly greater in the years 2016–2018 (range: 81.2%–85.7%) in comparison with 2015 (72.8%) (Mantel-Haenszel chi-square = 253.3, P < 0.0001) (Table 2). For all years, proportionally more worms were contained in areas under more intensive surveillance (Levels 1 or 2) compared to areas under less intensive surveillance (Level 3) (P < 0.0005 for all years and surveillance levels). Similarly, proportionally more dogs in Levels 1 and 2 were contained in 2018 in comparison with years past (Mantel-Haenszel chi-square = 66.3, P < 0.0001), and dogs were more likely to be contained in areas under the greatest intensity of surveillance (Table 3).

**Table 2:** Proportion of worms contained by surveillance level, 2015–2018.

			Number and Proportion of Contained Worms				
	Total Worms	Total Contained	Level 1	Level 2	Level 3		
	N	n (%)	n (%)	n (%)	n (%)	χ^2^	P^†^
2015*	981	714 (72.8)	NA	NA	NA	NA	
2016	2019	1639 (81.2)	1296 (85.7)	253 (75.8)	90 (52.0)	**123.1**	**<0.0001**
2017	1386	1162 (83.8)	914 (84.9)	231 (82.8)	17 (56.7)	**17.4**	**<0.0005**
2018	1962	1682 (85.7)	1608 (87.9)	52 (88.1)	22 (30.1)	**191.5**	**<0.0001**

Bold indicates statistical significance.

*Surveillance data by level were not available for the year 2015.

^†^Chi-square test for differences in the proportion of dogs contained by surveillance level within each year.

**Table 3:** Proportion of infected dogs contained by surveillance level, 2015–2018.

			Number and Proportion of Contained Dogs				
	Total Dogs	Total Contained	Level 1	Level 2	Level 3		
	N	n (%)	n (%)	n (%)	n (%)	χ^2^	P^†^
2015*	503	314 (62.4)	NA	NA	NA	NA	
2016	1011	740 (73.2)	616 (78.5)	101 (63.9)	23 (33.8)	**71.8**	**<0.0001**
2017	817	677 (82.9)	536 (82.3)	137 (88.4)	4 (36.4)	**20.2**	**<0.0001**
2018	1040	837 (80.5)	808 (83.0)	18 (75.0)	11 (25.6)	**87.0**	**<0.0001**

Bold indicates statistical significance.

*Surveillance data by level were not available for the year 2015.

^†^Chi-square test for differences in the proportion of dogs contained by surveillance level within each year.

Approximately 94% of lesions caused by *D*. *medinensis* appeared on dogs’ legs, consistent with typical presentation in humans. On average, infected dogs had 1.9 worms (SD: 2.2), and the distribution of worms per dog was highly aggregated and over dispersed (skewness = 14.9, kurtosis = 447.2), with a range of 1 to as many as 79. A slight majority of infected dogs were male (60.2%), and the average age of infected dogs was approximately 30 months (median: 24 months). The age of infected dogs identified in 2015 was significantly greater compared to infected dogs identified in 2016, 2017, and 2018 (S3 Table) (Kruskal-Wallis = 9.8, P = 0.02). Median dog age also varied significantly by region for all years (2015–2018) (S4 Table).

When asked about the dog’s infection history, owners reported a similar worm in a previous calendar year in 12.6% of 3,371 dogs. The proportion of dogs that had a history of infection in a previous year increased over time—this difference was most notable between 2016 and 2017 (Mantel-Haenszel chi-square = 18.8, P < 0.0001) (Table 4). Approximately 5% of owners of infected dogs reported a similar worm within the same year (that was not detected by the surveillance system). Among dogs with multiple worms (n = 1,298), the average time between emergence events between the first and second worm was 17.9 days. For 29 dogs, the number of days between emergence of the first and second worm was > 120 days. Dog owners reported that the majority of infected dogs were primarily used for guarding homes (92%, data only collected for 2018). Approximately 40% of infected dogs were used for hunting (data collected in 2017 and 2018), and about one-third of infected dogs were used for both purposes.

**Table 4:** Owner-reported history of previous Guinea worm-like illness in dogs infected with *Dracunculus medinensis* in Chad, 2015–2018.

	Canine Infection History					
	Previous Year* n = 406			Same Year*^†^ n = 159		
Year	n (%)	χ^2^	P	n (%)	χ^2^	P
2015	45 (9.0)			51 (10.2)		
2016	82 (8.2)			53 (5.3)		
2017	137 (16.8)			27 (3.3)		
2018	142 (13.7)			28 (2.7)		
		**18.8**	**<0.0001**		**38.8**	**<0.0001**

Bold indicates statistical significance.

*Missing values for previous year = 11, missing values for same year = 9.

^†^Only includes dogs that had worms not detected by the surveillance system (as reported by owner).

Rumors regarding dog infections increased notably over time, with 504 rumors in 2015, 2,154 rumors in 2016, 2,763 rumors in 2017, and 15,511 rumors in 2018.

### Owners of infected dogs

About one-third of owners of infected dogs reported owning just one dog (35.0%), with a mean of 2.3 dogs per owner. A plurality of dogs was owned by people of the Sara Kaba ethnicity (25.8%). Although 74.0% of dogs were reported to reside in fishing villages, only 17% of dogs were owned by people who were fishers or fish vendors. Farming-related occupations were reported among 73.5% of dog owners; hunting-related occupations were reported among 2.1% of dog owners (see S5 Table for a detailed breakdown of owner occupations).

### Spatial and temporal distribution of canine cases

**Fig 4:**
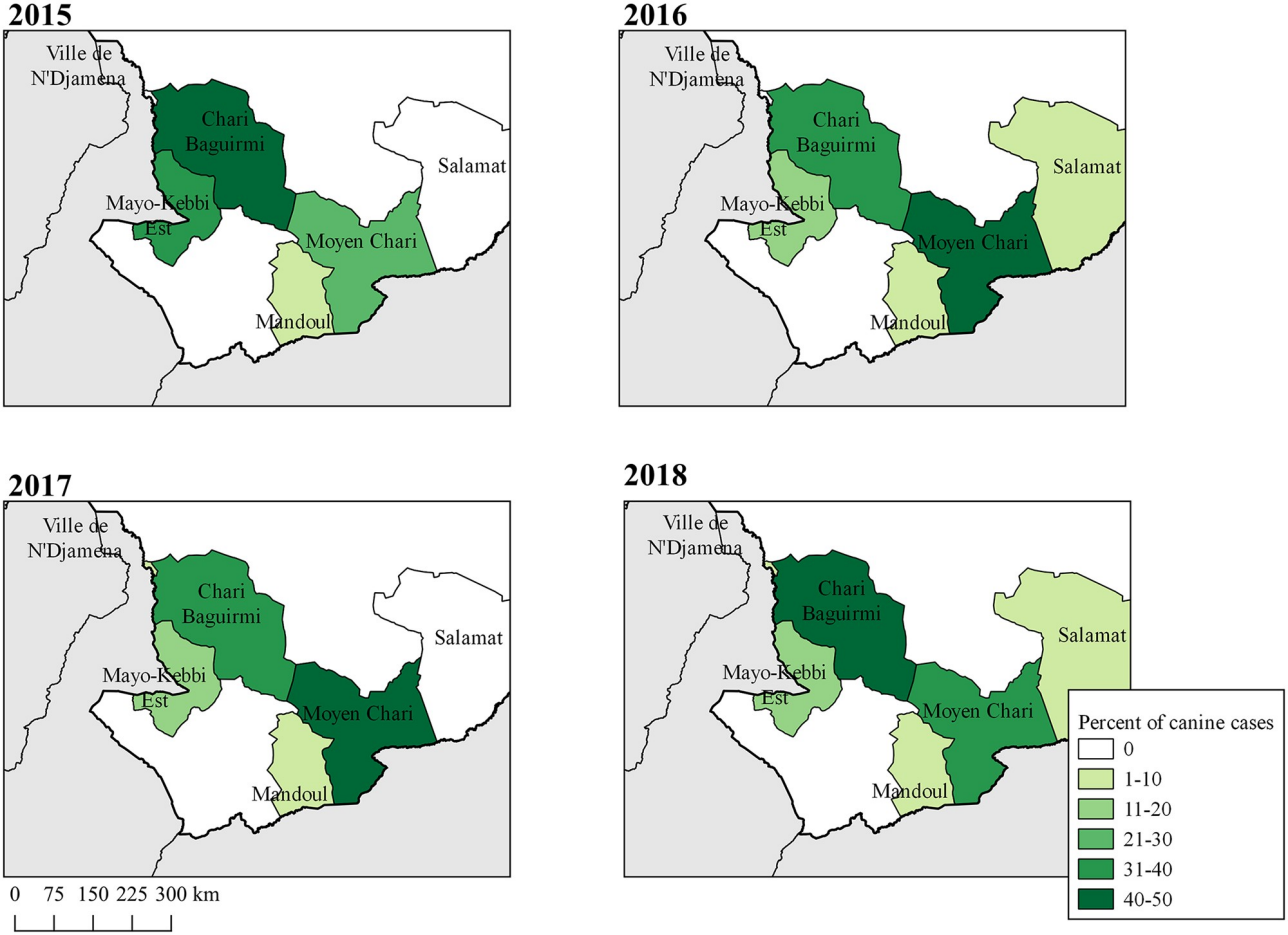
Percent of canine Guinea worm cases by region within Chad, 2015–2018. The majority of canine cases were concentrated in Moyen Chari and Chari Baguirmi Regions for all years of study.

The majority of canine *D*. *medinensis* infections (80%) occurred in just two of Chad’s 22 regions, Moyen Chari Region (southeast, 41.9%) and Chari Baguirmi Region (northwest, 38.1%) (Fig 4). Excluding regions in which few canine cases (<10) were recorded, the number of canine cases differed significantly by year and region (chi-square = 196.6, P < 0.0001). In 2016 and 2017 most cases derived from Moyen Chari Region, whereas in 2015 and 2018, more canine cases were from Chari Baguirmi Region.

**Fig 5:**
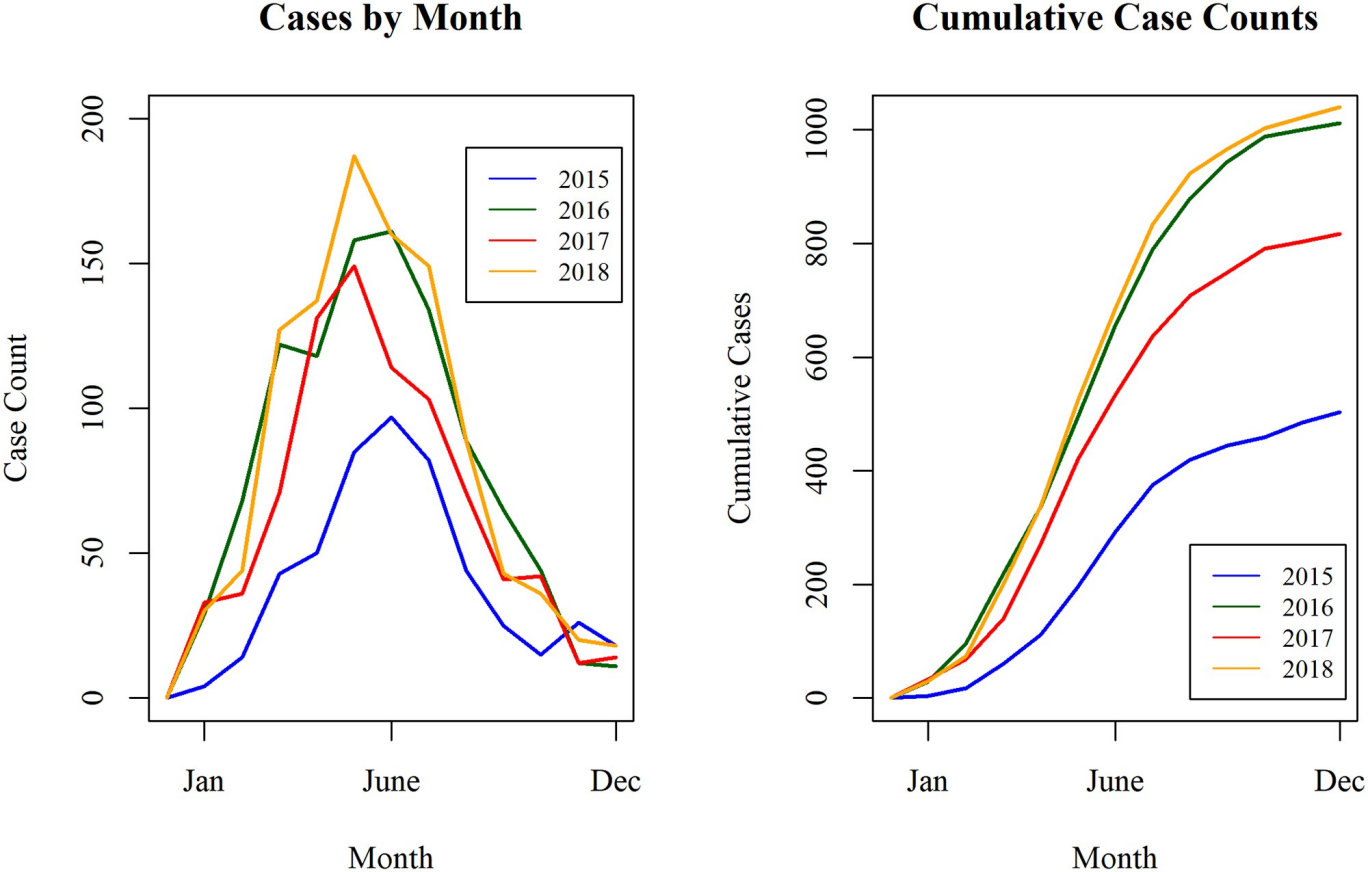
Canine Guinea worm cases by month in Chad, 2015–2018. Canine cases were most abundant during the months March through August; more cases were detected by the surveillance system over time.

The total number of canine cases detected more than doubled from 2015 to 2016 (503 canine cases in 2015 to 1011 canine cases in 2016). The number of canine cases fell in 2017 to 817, and then rose again in 2018 to 1,040. A strong seasonal pattern was observed: for each year the peak month of identified cases was June, with 78.5% of canine cases occurring during the months March through August (Fig 5). This pattern remained consistent for most regions within Chad, with the exception of Mandoul Region, in which canine cases were highest in the months October-December (S1 Fig).

## Discussion

Although the mechanisms driving canine *D*. *medinensis* infection are poorly understood, data collected through routine surveillance are fundamental to deciphering this mystery. Here, we discuss the increase in canine cases over time and the recurrence of infections in dogs, and consider the limitations of this analysis.

As noted elsewhere [1, 27], we observed an increase in the number of canine cases since the initial detection of Guinea worm in dogs in Chad in 2012. This could either be attributable to improved surveillance or ecologic changes that favor *D*. *medinensis* transmission. It is possible that both factors are responsible, but we are unable to disentangle the effects of surveillance bias vs. underlying epidemiology using surveillance data alone. A population genetics analysis showed a large, stable parasite population in Chad with no signatures of genetic bottlenecks or population expansions over time [28], lending support to the hypothesis that improved surveillance is the primary driver of increased canine cases. (However, this study was conducted on samples collected 2014–2016, and an analysis on more recent samples would be required to determine whether true population expansions have occurred during 2015–2018.) Further, over the past few years CGWEP has greatly expanded surveillance efforts (2,898 volunteers in 1,015 villages in 2015 to 6,427 volunteers in 1,895 villages in 2018) and invested in veterinary/animal health training courses (18 courses conducted for 172 field staff in 2018). Anecdotally, we have observed that the training courses have led to improved relationships between dogs and people (e.g., dogs are more open to being approached and touched by people), which may have resulted in better canine case detection. The relationship between the number of rumors of potential cases and actual cases detected from a given village/geographic area could be used to further explore the strength of the CGWEP surveillance system. Indeed, the CGWEP documented greater numbers of rumors in 2018 compared to years past [29], with the intention of bolstering canine case-finding.

Our results showed evidence of repeated infections in the same dog, both within and between years. Similar findings have been documented in Ghana in an analysis of human risk factors—the greatest relative risk was observed among individuals with a Guinea worm infection in the previous year [30]. Recurrent infection might relate to either underlying biological risk factors in the definitive host, or to risky behaviors repeated over time. Dogs are highly associated with humans, leading us to consider the relationship between canine *D*. *medinensis* infection and anthropogenic activity. For example, are dogs from certain households more likely to experience repeated infections over time? And if so, what human activities are influencing canine infection risk? Although the surveillance system does not currently track individual dogs over time, plans are underway to use fuzzy matching approaches to identify dogs and owners that appear in the data multiple instances over the course of different years, and to explore factors associated with recurrent infection. In the future, microchipping of a cohort of dogs may improve our ability to track individual animals, thereby revealing more information about risk of infection in dogs over time. To elucidate questions related to canine infection risk, the CGWEP has invested in a robust, multidisciplinary research agenda that includes work related to *D*. *medinensis* population genetics [28], experimental and field investigations of paratenic and transport hosts [31, 32], among others.

Some limitations of this analysis should be noted. It is unclear as to whether the containment data truly measure the surveillance system’s effectiveness in responding to canine cases. Of most concern are worms that may have emerged for a given dog *prior* to the surveillance system’s detection of that dog–approximately 5% of dog owners reported a history of Guinea worm-like infection in their dog within the same year, suggesting an issue with surveillance system sensitivity. Secondly, the CGWEP does not currently track individual dogs over time, therefore limiting our ability to determine whether the same dog presented with multiple worms in distinct instances. This may result in the overreporting of canine cases within years. Field staff rely on dog name, owners/households, and relationships with the communities to properly identify dogs; this method is subject to errors in dog identification. Dog owners also may change from year to year, further complicating efforts at identifying the same dog over time. The reported age of the dog may not be accurate because many dog owners do not keep track of their dogs’ ages, particularly after the dog has reached maturity. This leads us to question whether heterogeneities in dog age over time and space are real, or whether they are an artifact of surveillance. Lastly, the cash reward system has likely influenced reporting of cases over time [33]. A more thorough follow-up analysis could help explain the relationship between the cash incentive program and the sensitivity of the surveillance system, and as surveillance expanded to new geographic areas within the country since the inception of the CGWEP.

Surveillance data show that canine Guinea worm cases cluster in terms of space (Moyen Chari and Chari Baguirmi Regions), time (most cases occur March-August), and within canine hosts (with recurrent infections). If these patterns are reflective of underlying transmission biology, interventions may be targeted to these regions, at certain times of year, and even to certain households and dogs. As the world’s largest active community-based surveillance system that simultaneously searches for disease in both humans and animals alike, the CGWEP novel surveillance approaches presented here may inform efforts directed towards other zoonoses (e.g., rabies in particular is a known problem in Chad [34, 35]). (Other examples of active surveillance for zoonoses have focused predominantly on livestock—for example, influenza A virus among pigs in the United States and Taiwan [36, 37], and bovine spongiform encephalopathy screening among fallen cattle in Europe [38].) Methods of interest may include data flow processes, active case finding in dogs, and community engagement in identifying and reporting rumors.

## Supporting information

S1 Appendix Details of the CGWEP surveillance system. More information about data flow, data validation, reporting, worm extraction, interventions, and transition between levels of surveillance is described.
(DOCX)

S2 Appendix CGWEP animal infection form. The 2018 version of the form used to collect information about infected dogs.
(PDF)

S1 Table Cash reward structure, 2015–2018. The cash reward for various reporting events is shown. The cash reward is the same for all levels of surveillance throughout Chad. In humans, the cash reward is disbursed only after the worm has been laboratory-confirmed as Guinea worm. For dogs, the cash reward is disbursed when a regional supervisor visually inspects the worm.
(DOCX)

S2 Table Number and proportion of specimens received by the CDC Parasitic Diseases Laboratory confirmed as *D*. *medinensis* by year. Of the 22 worm specimens (extracted from dogs) tested, 13 were confirmed as *D*. *medinensis*.
(DOCX)

S3 Table Median and mean ages of dogs infected with *Dracunculus medinensis* in Chad by year, 2015–2018. In 2015, the mean age of dogs detected was older in comparison with other years.
(DOCX)

S4 Table Median and mean ages of dogs infected with *Dracunculus medinensis* in Chad by year and region, 2015–2018. Mandoul Region had the oldest infected dogs for the years 2015–2017. In 2018, however, this region had the youngest infected dogs.
(DOCX)

S5 Table Frequencies of select occupations of owners of dogs infected with *Dracunculus medinensis* in Chad, 2015–2018. Fishing, hunting, and farming occupations are listed first for emphasis, due to possible exposures to fish or frogs (which may serve as paratenic or transport hosts).
(DOCX)

S1 Fig Canine Guinea worm cases by region in Chad, 2015–2018. In most regions within Chad the occurrence of canine cases peaked in the month of June. In Mandoul Region, canine cases peaked in October.
(TIFF)
