# Supplementary material for: Guinea worm in domestic dogs in Chad: A description and analysis of surveillance data
Source: PLoS Negl Trop Dis. 2020 May 28;14(5):e0008207. doi: 10.1371/journal.pntd.0008207 (PMC7255611; doi:10.1371/journal.pntd.0008207)
Supplement: S1 Appendix — More information about data flow, data validation, reporting, worm extraction, interventions, and transition between levels of surveillance is described. (DOCX) [file pntd.0008207.s001.docx]

**S1 Appendix. Details of the CGWEP surveillance system.**

**Data flow**Data are initially captured on paper forms; separate human and animal forms exist to gather information about interventions, rumors, and cases investigated. Volunteers in each village under active surveillance make visits to households to detect GWD symptoms among humans and animals, and in turn, notify field supervisors who help investigate all rumors and cases and complete paper reporting forms. Rumors/cases are investigated within 24 hours of notification more than 97% of the time. Paper forms are shipped to regional CGWEP data management hubs (Sarh, Guelendeng, and N’Djamena) where CGWEP data managers check for validity and enter the data into Microsoft Excel spreadsheets. The Excel files are then sent via email to the CGWEP headquarters in N’Djamena where the lead Data Manager combines and validates the data again. Paper forms are photocopied in the regional hubs and paper copies are sent to the district hospitals while original versions are shipped to N’Djamena for final validity checks (if needed) and archiving.

**Data validation procedures**

Data on rumors and canine cases are validated both in the field and at the national level. For rumors of canine cases, each month, field supervisors cross-check information entered on paper forms with the electronic data entered into Excel spreadsheets. For canine cases, when the electronic data are forwarded to the lead Data Manager in N’Djamena (weekly), additional cross-checks are conducted (i.e., do the number of villages listed in a village-level dataset match those that are listed at the worm and dog levels?). Possible data entry errors are identified by checking the electronic datasets for extreme or missing values and spelling mistakes (e.g., village names); possible duplicate entries are also identified and corrected on a weekly basis, and then again at the end of each month. Discrepancies identified at the national level are flagged and returned to field supervisors for correction. Finally, corrected data are re-submitted to the national CGWEP program in N’Djamena for further checking and for generating summary reports (described below).

Periodic trainings with field agents are conducted to minimize potential errors and misunderstandings of variables on the forms used for data collection. Forms are updated yearly with input from the field staff and CGWEP staff based in N’Djamena.

**Reporting**

Summarized data reports are sent weekly to the Integrated Epidemiological Surveillance Service of the Ministry of Public Health (MOPH) and this data is presented every Wednesday at the MOPH. On the 18th day of the month, the CGWEP Data Manager in N’Djamena sends the compiled and summarized data to CGWEP leadership. On the 20th day of the month, the CGWEP Coordinator (head of the national program) shares the data with relevant partners, including the World Health Organization Guinea Worm office and The Carter Center staff based in Atlanta, Georgia.

**Interventions**

Health education about Guinea worm disease prevention in humans and in dogs/cats is a crucial component of the CGWEP. Health education efforts are focused on preventing contamination of water sources; for canine cases, this entails tethering of dogs to prevent them from entering water, and for humans this entails encouraging patients to remain at health centers.

In addition, Abate® (temephos) is applied to kill copepods and interrupt the transmission cycle. Temephos is applied to the source(s) of contaminated water, if known. In 2018, select villages (with > 5 dog infections in the current or previous year) began receiving prescriptive treatment, in which Abate is pre-emptively applied to all water sources in and around the locality. (In 2019, this strategy has expanded to include all villages reporting > 1 dog infection in the current or previous year.) Additional interventions are also carried out, including advocacy for the provision of safe water, burial of fish guts, tethering of animals, and promotion of the cash reward system. Cloth and pipe filters are also distributed in villages with > 1 infection and no borehole.

**Transition between levels of surveillance**The transition between surveillance levels is based on the occurrence of *D. medinensis* infections in the area. Historically, a village was upgraded to Surveillance Level 1 when indigenous transmission of *D. medinensis* was confirmed (in humans or in animals) within a single year. Recently, however, the CGWEP has intensified surveillance efforts, and in 2019, any village with > 1 case of Guinea worm (in humans or in animals) will be included in Level 1. Surveillance intensity is only scaled down once transmission is interrupted, defined as zero indigenous infections in humans or animals reported for 14 consecutive months or more, with minimal risk of importation.

**Community involvement**

The CGWEP relies on an extensive network of volunteers to identify cases in humans and in animals, and to provide communities with health education. Village volunteers receive incentives to encourage their contributions to the CGWEP surveillance system, such as participation in regular trainings (at least three times per year), and receipt of t-shirts and other health education materials. Volunteers also receive a 10,000 CFA transportation allowance at every training.

A minimum of two volunteers are assigned to each village, depending on the overall population size of the village. In most surveillance locations there are just two volunteers, but in a few very large villages there can be up to 130 volunteers. Volunteers are equally represented by women and men to ensure better communication across genders.

In recent years, a new program called ‘the dog police,’ has been implemented in some endemic areas to engage youth in identifying dogs with Guinea worm infections. Dog police were selected on the basis of willingness of youths to participate, and verbal assent from their parents. It is an entirely voluntary activity, and provides kids with the opportunity to learn about Guinea worm and engage with the program. A “youth leader” is first identified by the CGWEP field staff. This this person usually demonstrates keen interest in Guinea worm, and has influence over their peers in the community. Once identified, the youth leader is taught about Guinea worm identification in dogs and reporting procedures; they also encourage others in the community to check dogs on a regular basis.
