# Supplementary material for: Guinea worm in domestic dogs in Chad: A description and analysis of surveillance data
Source: PLoS Negl Trop Dis. 2020 May 28;14(5):e0008207. doi: 10.1371/journal.pntd.0008207 (PMC7255611; doi:10.1371/journal.pntd.0008207)
Supplement: S2 Appendix — The 2018 version of the form used to collect information about infected dogs. (PDF) [file pntd.0008207.s002.pdf]

Programme National d'Éradication du Ver de Guinée au Tchad

Formulaire d'infection - Ver chez les animaux

I. Localité:

1. Région: ______________________________ **2.** District: ____________________________

3. Zone: ________________________________ **4.** Village: ____________________________

5. Quartier: _____________________________ **6.** GPS: N_____________ E ____________

7. Niveau de surveillance: ☐ Niveau 1 ☐ Niveau 2 ☐ Niveau 3 ☐ Nomade

II. Données de l'animal

1. Type d'animal: _____________________ **2.** Population de chiens dans le village: _____

3. Nom du propriétaire de l'animal _____________________________ 4. N°de tel: ________________

5. Ethnie du propriétaire: __________ **6.** Occupation(s) du propriétaire:____________________

7. Nom de l'informateur________________ **8**. Occupation de l'informateur:________________________

9. Age de l'animal ______ **10.** Sexe de l'animal _______ **11.** Nom de l'animal ____________________

12. L'animal habitait où l'année dernière? __________________

13. Nb. Total de chien du propriétaire: ______

14. Avant ce(s) ver(s), l'animal a déjà eu un ver cette année (2018)? ☐ Oui ☐ Non

15. Cet animal a eu un ver l'année passée (2017)? ☐ Oui ☐ Non

16. Ce chien est utilisé pour:
- La chasse? ☐ Oui ☐ Non
- Gardinage? ☐ Oui ☐ Non
- Autre? _____________________

III. Critères d'isolement remplis:

1 -

a. L'animal a-t-il été attaché **avec une chaine** avant qu'il y ait la plaie: ☐ Oui ☐ Non

b. Date d'attachement **avec une chaine et 2 cadanas** : _________________________________

c. Cet animal est resté attaché jusqu'à la cicatrisation de la plaie: ☐ Oui ☐ Non

2 - Animal investigué dans les 24 heures après l'émergence du ver: ☐ Oui ☐ Non

3 - L'animal a été observé par un agent de santé/VG dans 7 jours: ☐ Oui ☐ Non

4 -

a**.** Contamination probable d'une source d'eau: ☐ Oui ☐ Non

b**.** Sources d'eau probablement contaminées: ____

c. Nombre de sources d'eau contaminées? _____________

IV. Remplir les dates pour chaque ver (pour plus de 5 vers, remplir une 2eme fiche)

Nb. ver	Date du:						
	Détection*	Notification	Par qui?	Investigation	Par qui?	Emergence	Prélèvement
1							
2							
3							
4							
5							

**La Date de Detection correspond au premier jour où une personne à vu les symptomes du VG chez l'animal (ampoule, gonflement, plaie ou ver)*

Localisation du ver (les nombres du ver correspondent aux lignes dans le tableau des dates):

1. ____
2. ____
3. ____
4. ____
5. ____

V. Autres informations/ Observations:

1. Source d'eau potable dans le village: ☐ oui ☐ non

2. Présence des ASVs dans le village: ☐ oui ☐ non

VI. Mesures prises: (Cochez tout ce qui s'applique)

Attacher l'animal avec une corde et le soigner ☐
Donner l'éducation au propriétaire ☐
Donner l'éducation au chef et aux villageois ☐
Notifier l'équipe du VG (AR, SASV, CT, RCS) ☐
Faire l'immersion contrôlée ☐
Traitement de mare contaminée ☐
Filtres distribués ☐
Echantillon pris ☐

Commentaires/Observations:

Rempli par/ fonction: _____________________________ ***Contact:*** ___________________

VII. Rempli par CT___________________________

Date de soumission de la fiche électronique:

Date de soumission de la version papier:

No. des échantillons pris:

Date d'envoie de l'échantillon à NDJ:

Animal détecté comme rumeur: OUI/NON

Date de détection de la rumeur:

Le ver est: isolé / en cours d'isolation / non isolé

Non isolé pour les raisons suivantes: ____________________________________

Observations:
