# Supplementary material for: Guinea worm in domestic dogs in Chad: A description and analysis of surveillance data
Source: PLoS Negl Trop Dis. 2020 May 28;14(5):e0008207. doi: 10.1371/journal.pntd.0008207 (PMC7255611; doi:10.1371/journal.pntd.0008207)
Supplement: S1 Table — The cash reward for various reporting events is shown. The cash reward is the same for all levels of surveillance throughout Chad. In humans, the cash reward is disbursed only after the worm has been laboratory-confirmed as Guinea worm. For dogs, the cash reward is disbursed when a regional supervisor visually inspects the worm. (DOCX) [file pntd.0008207.s003.docx]

**S1 Table. Cash reward structure, 2015-2018.**

|  | **Cash Reward Amount*** | |
| --- | --- | --- |
| **Event** | **Self-report** | **Informant report** |
| Laboratory-confirmed worm in **human** | 50,000 | 25,000/ 25,000^†^ |
| **Dog** worm *before* worm emergence | Dog owner receives 10,000 & 3 bars of soap | No reward for informant |
| **Dog** worm *after* worm emergence | Dog owner receives 10,000 | No reward for informant |
| Signs and symptoms in **dogs** reported | Dog owner receives 3 bars of soap | No reward for informant |

*Currency in Central African Francs (CFA). As of December 2019, the exchange rate was approximately 1 USD = 592 CFA
^†^Reward is split between the patient and the informant; each receives 25,000 CFA.
