# Supplementary material for: Guinea worm in domestic dogs in Chad: A description and analysis of surveillance data
Source: PLoS Negl Trop Dis. 2020 May 28;14(5):e0008207. doi: 10.1371/journal.pntd.0008207 (PMC7255611; doi:10.1371/journal.pntd.0008207)
Supplement: S2 Table — Of the 22 worm specimens (extracted from dogs) tested, 13 were confirmed as D. medinensis. (DOCX) [file pntd.0008207.s004.docx]

| **Year** | **Confirmed Specimens** | **(%)** |
| --- | --- | --- |
| 2015 | 1 | (100) |
| 2016 | 5 | (50) |
| 2017 | 1 | (50) |
| 2018 | 6 | (66.7) |

**S2 Table. Number and proportion of specimens received by the CDC Parasitic Diseases Laboratory confirmed as *D. medinensis* by year*.***
