# Supplementary material for: Guinea worm in domestic dogs in Chad: A description and analysis of surveillance data
Source: PLoS Negl Trop Dis. 2020 May 28;14(5):e0008207. doi: 10.1371/journal.pntd.0008207 (PMC7255611; doi:10.1371/journal.pntd.0008207)
Supplement: S3 Table — In 2015, the mean age of dogs detected was older in comparison with other years. (DOCX) [file pntd.0008207.s005.docx]

**S3 Table. Median and mean ages of dogs infected with *Dracunculus medinensis* in Chad by year, 2015-2018.**

|  |  |  |  |  |  |
| --- | --- | --- | --- | --- | --- |
| **Year** | **n** | **Median age (months)** | **Mean age (months)** | **Kruskal-Wallis** | **P** |
|  |  |  |  |  |  |
| 2015 | 479 | 24 | 32.7 |  |  |
| 2016 | 961 | 24 | 29.0 |  |  |
| 2017 | 797 | 24 | 29.0 |  |  |
| 2018 | 992 | 24 | 30.0 |  |  |
| Missing | 152 |  |  |  |  |
| **Total dogs** | 3371 |  |  | 9.8 | 0.02 |
