# Supplementary material for: Guinea worm in domestic dogs in Chad: A description and analysis of surveillance data
Source: PLoS Negl Trop Dis. 2020 May 28;14(5):e0008207. doi: 10.1371/journal.pntd.0008207 (PMC7255611; doi:10.1371/journal.pntd.0008207)
Supplement: S4 Table — Mandoul Region had the oldest infected dogs for the years 2015–2017. In 2018, however, this region had the youngest infected dogs. (DOCX) [file pntd.0008207.s006.docx]

| **All years** | | | | | |
| --- | --- | --- | --- | --- | --- |
| **Region** | **N** | **Median age (months)** | **Mean age (months)** | **Kruskal-Wallis** | **P** |
| Moyen Chari | 1397 | 24 | 30.8 |  |  |
| Chari Baguirmi | 1249 | 24 | 28.7 |  |  |
| Mayo-Kebbi Est | 504 | 24 | 30.2 |  |  |
| Mandoul | 142 | 36 | 34.6 |  |  |
| Other regions* | 13 |  |  |  |  |
| Missing | 66 |  |  |  |  |
| **Total dogs** | 3371 |  |  |  |  |
|  |  |  |  | 41.3 | < 0.0001 |
| **2015** | | | | | |
| **Region** | **N** | **Median age (months)** | **Mean age (months)** | **Kruskal-Wallis** | **P** |
| Moyen Chari | 116 | 24 | 32.5 |  |  |
| Chari Baguirmi | 201 | 36 | 34.1 |  |  |
| Mayo-Kebbi Est | 149 | 24 | 28.7 |  |  |
| Mandoul | 25 | 36 | 46.1 |  |  |
| Missing | 12 |  |  |  |  |
| **Total dogs** | 503 |  |  |  |  |
|  |  |  |  | 25.8 | < 0.0001 |
| **2016** | | | | | |
| **Region** | **N** | **Median age (months)** | **Mean age (months)** | **Kruskal-Wallis** | **P** |
| Moyen Chari | 507 | 24 | 29.6 |  |  |
| Chari Baguirmi | 298 | 24 | 27.5 |  |  |
| Mayo-Kebbi Est | 118 | 24 | 27.2 |  |  |
| Mandoul | 62 | 36 | 34.8 |  |  |
| Other regions* | 1 |  |  |  |  |
| Missing | 25 |  |  |  |  |
| **Total dogs** | 1011 |  |  |  |  |
|  |  |  |  | 24.6 | < 0.0001 |
| **2017** | | | | | |
| **Region** | **N** | **Median age (months)** | **Mean age (months)** | **Kruskal-Wallis** | **P** |
| Moyen Chari | 369 | 24 | 32.1 |  |  |
| Chari Baguirmi | 304 | 24 | 27.0 |  |  |
| Mayo-Kebbi Est | 99 | 24 | 29.8 |  |  |
| Mandoul | 34 | 36 | 32.5 |  |  |
| Other regions* | 1 |  |  |  |  |
| Missing | 10 |  |  |  |  |
| **Total dogs** | 817 |  |  |  |  |
|  |  |  |  | 27.1 | < 0.0001 |
| **2018** | | | | | |
| **Region** | **N** | **Median age (months)** | **Mean age (months)** | **Kruskal-Wallis** | **P** |
| Moyen Chari | 405 | 24 | 30.7 |  |  |
| Chari Baguirmi | 446 | 24 | 28.3 |  |  |
| Mayo-Kebbi Est | 138 | 24 | 34.7 |  |  |
| Mandoul | 21 | 24 | 24 |  |  |
| Other regions* | 11 |  |  |  |  |
| Missing | 19 |  |  |  |  |
| **Total dogs** | 1040 |  |  |  |  |
|  |  |  |  | 14.2 | 0.0027 |

**S4 Table. Median and mean ages of dogs infected with *Dracunculus medinensis* in Chad by year and region.**

*Other regions included N’Djamena, Tandjile, and Salamat. Dogs from these regions were excluded from the statistical test because of low counts.
