# Supplementary material for: Guinea worm in domestic dogs in Chad: A description and analysis of surveillance data
Source: PLoS Negl Trop Dis. 2020 May 28;14(5):e0008207. doi: 10.1371/journal.pntd.0008207 (PMC7255611; doi:10.1371/journal.pntd.0008207)
Supplement: S5 Table — Fishing, hunting, and farming occupations are listed first for emphasis, due to possible exposures to fish or frogs (which may serve as paratenic or transport hosts). (DOCX) [file pntd.0008207.s007.docx]

**S5 Table. Frequencies of select occupations of owners of dogs infected with *Dracunculus medinensis* in Chad, 2015–2018.**

|  |  |  |
| --- | --- | --- |
| **Occupations and combinations** | **n** | **(%)** |
|  |  |  |
| Farmer only | 2254 | (66.9) |
| Fisherman only | 410 | (12.2) |
| Farmer + Fisherman | 159 | (4.7) |
| Farmer + Hunter | 60 | (1.8) |
| Hunter only | 7 | (0.2) |
| Farmer + Fisherman + Hunter | 4 | (0.1) |
| Fisherman + Hunter | 0 | -- |
| Housewife | 234 | (6.9) |
| Teacher | 48 | (1.4) |
| Merchant | 20 | (0.6) |
| Security guard | 14 | (0.4) |
| Other occupation* | 120 | (3.6) |
| Missing | 41 | (1.2) |
| **Total dog owners** | 3371 | (100) |

*Other occupations include technicians, sugar cane workers, retirees, nurses, pastors, construction workers, policemen, children, drivers, military service members, social workers, tailors, students, factory workers, government officials, and drivers. Each of these occupations represented less than 1% (<9 individuals) of all dog owners.
