# Supplementary figures and images for: Guinea worm in domestic dogs in Chad: A description and analysis of surveillance data
Source: PLoS Negl Trop Dis. 2020 May 28;14(5):e0008207. doi: 10.1371/journal.pntd.0008207 (PMC7255611; doi:10.1371/journal.pntd.0008207)

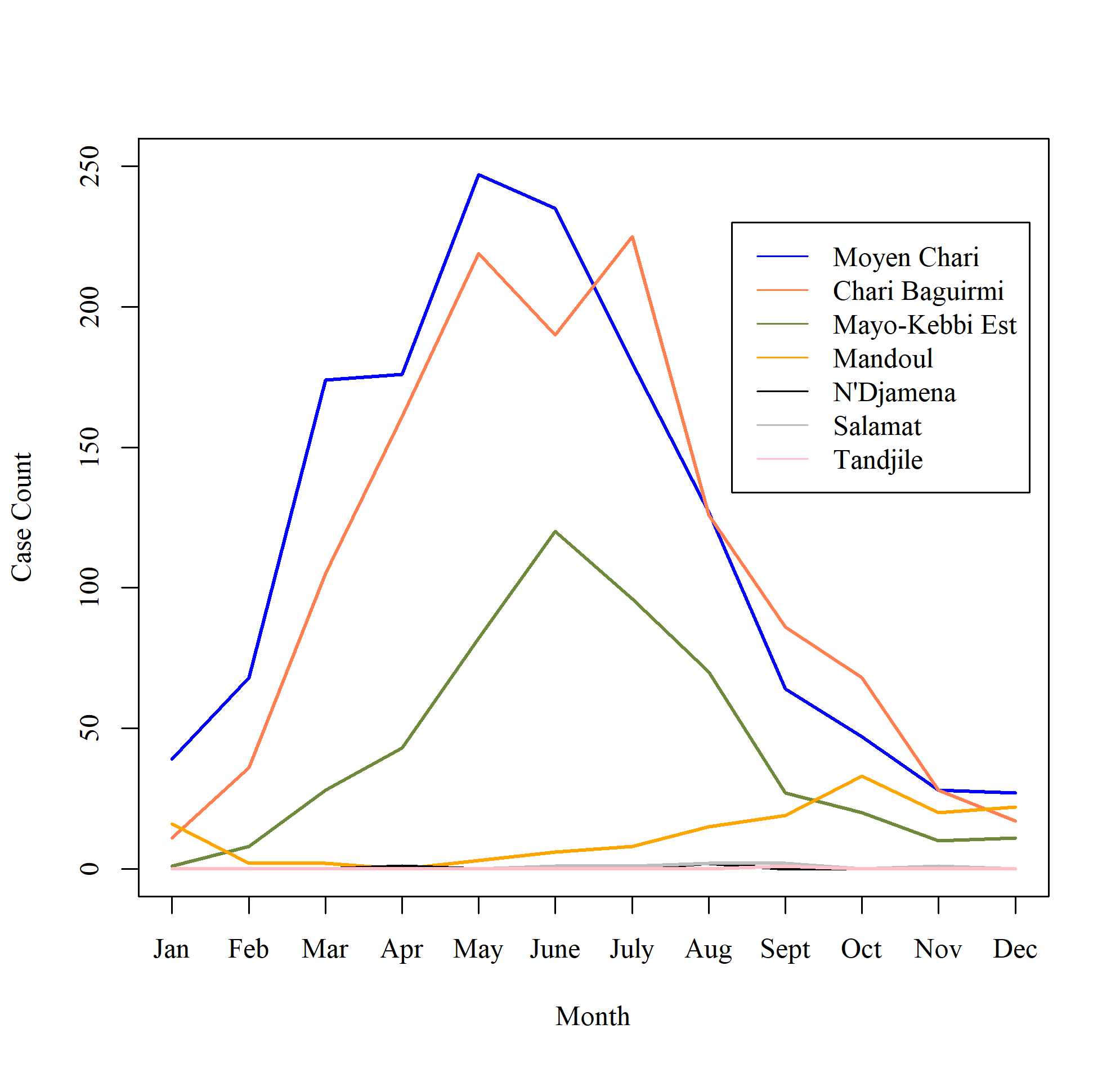

Supplement: S1 Fig — In most regions within Chad the occurrence of canine cases peaked in the month of June. In Mandoul Region, canine cases peaked in October. (TIFF) [file pntd.0008207.s008.tiff]
